# Sex Ratio Elasticity Influences the Selection of Sex Ratio Strategy

**DOI:** 10.1038/srep39807

**Published:** 2016-12-23

**Authors:** Yaqiang Wang, Ruiwu Wang, Yaotang Li, Zhanshan (Sam) Ma

**Affiliations:** 1 Institute of Mathematics and Information Science, Baoji University of Arts and Sciences, Baoji, Shaanxi, China; 2 Computational Biology and Medical Ecology Lab, Theoretical and Experimental Ecology Lab, State Key Laboratory of Genetic Resources and Evolution, Kunming Institute of Zoology, Chinese Academy of Science, Kunming, Yunnan, China; 3 Center for Ecological and Environmental Sciences, Key Laboratory for Space Bioscience & Biotechnology, Northwestern Polytechnical University, Xi’an, Shaanxi, China; 4 School of Mathematics and Statistics, Yunnan University, Kunming, Yunnan, China

## Abstract

There are three *sex ratio strategies* (SRS) in nature—male-biased sex ratio, female-biased sex ratio and, equal sex ratio. It was R. A. Fisher who first explained why most species in nature display a sex ratio of ½. Consequent SRS theories such as Hamilton’s *local mate competition* (LMC) and Clark’s *local resource competition* (LRC) separately explained the observed deviations from the seemingly universal 1:1 ratio. However, to the best of our knowledge, there is not yet a unified theory that accounts for the mechanisms of the three SRS. Here, we introduce the *price elasticity theory* in economics to define *sex ratio elasticity* (SRE), and present an analytical model that derives three SRSs based on the following assumption: simultaneously existing competitions for both resources A and resources B influence the level of SRE in both sexes differently. Consequently, it is the difference (between two sexes) in the level of their sex ratio elasticity that leads to three different SRS. Our analytical results demonstrate that the elasticity-based model not only reveals a highly plausible mechanism that explains the evolution of SRS in nature, but also offers a novel framework for unifying two major classical theories (*i.e.,* LMC & LRC) in the field of SRS research.

The *sex ratio* is usually defined as the proportion of males in a population, and it can further be classified as *the primary, secondary*, and *tertiary sex ratio*. We are concerned with the first one, which refers to the ratio of at time of conception^1^,^2^. The *sex ratio strategy* (SRS) is the sex ratio pattern that is exhibited by a species in nature, and its variation can directly affect the structure of population and its mating system^2^,^3^,^4^. In nature, different species choose three different sex ratio strategies: male-biased sex ratio, female-biased sex ratio and, equal sex ratio, depending on sex ratio being greater than, equal, or less than ½, in terms of the proportion of male offspring in the whole population. In spite of the extensive studies in the field since Darwin (1859) and Fisher (1930), the evolution of SRS is still a hotly debated topic in evolutionary biology^5^,^6^.

In 1930, Fisher assumed that males and females are equally costly to produce, equal numbers of both sexes should be produced, leading to the sex ratio of ½^3^,^6^. Although it has recently been discovered that this theory was actually first put forward in the 19^th^ century by the German biologist Carl Düsing in his dissertation, who was among the first who resorted to mathematical modeling for solving evolutionary biology problems^3^,^7^, we propose the equal investment theory in this paper as presented by Fisher. An implicit assumption in Fisher’s *equal investment theory* is that there are no competitive or cooperative interactions among relatives^3^. Obviously, when populations are structured, competitive interactions between siblings could occur in each patch, such as, mate competition among male offspring, and resource competition among female offspring^3^,^8^,^9^.

Mate competition among male offspring in a structured population is termed as local mate competition (LMC), it was first introduced by W. D. Hamilton to explain extraordinary female-biased sex ratios observed in a variety of insects and mites^2^,^3^,^8^. Hamilton considered the mating system of diploid organisms, and showed that the evolutionary stable strategy (ESS) sex ratio (s*), or what he termed ‘unbeatable’ sex ratio, can be represented as: s* = (*n* − 1)/(2*n*), where *n* is the number of foundress per patch^8^. In 1979, Hamilton extended his original LMC theory for diploid to the case of haplo-diploid organism, and noted that inbreeding causes mothers to be relatively more related to their daughters than to their sons, which leads to a slightly more female biased sex ratio being favored^3^,^10^. LMC theory predicts female-biased sex ratio is an ESS when mating takes place locally and related male offspring compete for mates. However, W. D. Hamilton (1967, 1979) only explored the effect of competition among male offspring on the SRS, and resource competition among female offspring could also influence the SRS^3^,^9^.

In the study of African bush baby (*Galago crassicaudaus*), Clark (1978) found that during breeding season, female’s movement is restricted by her ‘responsibility’ for raising offspring. If male offspring (instead of female offspring) disperse from the natal site while female offspring stay local and compete with each other for resources (such as space, food), then local resource competition (LRC) among females can occur. From the observation, Clark (1978) proposed that female offspring compete for resources (such as space, food), but male offspring leave their birthplace to find new mates. Clark (1978) further postulated with mathematical modeling that female competition for resources can lead to male-biased SRS^3^,^9^.

In summary, existing LMC and LRC models, including their extensions addressed either the effect of competition among male offspring or that among female offspring on the ESS of sex ratio, *respectively*^11^,^12^,^13^,^14^,^15^,^16^,^17^. However, many field observations have discovered that mate competition among male offspring and resource competition (such as nest) among female offspring often occur simultaneously in nature^3^,^18^. Obviously, *mates* can also be considered as a *resource* different from food and shelters.

The simultaneous of these competitions might lead to the difference intensity between male competition and female competition. However, the difference intensity between male competition and female competition could lead to the difference of the sex ratio elasticity of male offspring survival rate and the sex ratio elasticity of female offspring survival rate, which may affect the selection of sex ratio strategy. The concept of elasticity (famous in economics) was first introduced by Wang *et al*. to measure the responsiveness of offspring survival rate to a change in reproductive allocation^19^. The sex ratio elasticity of male (female) survival rate is a measure used to show the responsiveness of male (female) survival rate to a change in sex ratio. It could be defined as the percentage change in male (female) survival rate divided by the percentage change in sex ratio, and this similar to elasticity concept in economics^20^.

Although the existing model do already incorporate the simultaneous mate competition among male offspring and resource competition among female offspring into a single framework^3^,^18^, to the best of our knowledge, in the existing literature, how the sex ratio elasticity of male (female) survival rate affects the SRS, which has never been studied before. Therefore, it is still a challenge to incorporate the LMC and LRC into a single framework based on the sex ratio elasticity, and to explore the effect of the sex ratio elasticity of male (female) survival rate on SRS.

In the present study, we construct a new sex ratio model, and assume that male offspring and female offspring compete for different resources, and male offspring compete for resource A (such as space, mates) and female offspring compete for resource B (such as nests), moreover, these competitions occur simultaneously in the mating system. Applying MacArthur’s product rules^21^. Our analysis reveals that the equilibrium sex ratio (ESR) depends on the *sex ratio elasticity* of the male offspring’s survival rate (SRE-MSR) and the *sex ratio elasticity* of the female offspring’s survival rate (SRE-FSR). Furthermore, we found that both the simultaneous existing competitions could create asymmetricity between males and females in their intensities of competitions. Moreover, the asymmetricity in the intensity can lead to the difference between the *sex ratio elasticity* of the SRE-MSR and SRE-FSR. Then, it is the difference in the sex ratio elasticity that influences the evolution of sex ratio strategy.

## The model

Considering a sexual species, which has discrete generations and their offspring remain their natal site. Assuming that male offspring and female offspring compete for different resources, and male offspring compete for resource A (such as space, mates) and female offspring compete for resource B (such as nests), i.e., there are two competitions occurring simultaneously.

Let *N* be the clutch size of the adult individual, *m* and *f* be the number of male offspring and the number of female offspring alive at breeding age respectively. We further assume that *r* is the proportion of male offspring, *i.e*., the sex ratio, in a clutch, *1* − *r* is the proportion of female offspring in the same clutch. *S*_*m*_ is the survival rate of male offspring, and *S*_*f*_ is the survival rate of female offspring.

Based on the above assumptions, the number of male offspring is


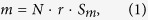


and the number of female offspring is


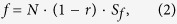


We further assume that the population is effectively infinite, and the brood’s sex ratio is determined by the maternal genotype. According to the *de facto* standard treatment in sex-ratio theory, the evolutionary stable strategy (ESS) maximizes the product of *m* × *f*, which is known as “MacArthur product rule” in the literature^4^,^15^,^16^.

From Equations (1) and (2), the product of *m* and *f* is given by


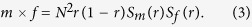


Since male offspring compete for resources A, the increase of their number (i.e. *r* increase) should result in the decrease of their survival rate. Similarly, since the competition for the resources B among female offspring, the decrease of their number (i.e., *r* increase) should lead to the increase of their survival rate. In other words, *S*_*m*_ and *S*_*f*_ should be the function of *r, i.e*., *S*_*m*_ = *S*_*m*_(*r*) and *S*_*f*_ = *S*_*f*_(*r*), and their derivatives should satisfy the following conditions: *dS*_*m*_/*dr* < 0 and *dS*_*f*_/*dr* > 0.

From (3), the product *m* × *f* is a function of the sex ratio *r*, and it achieves its maximal value with respect to *r* when *r* = r^*^, where r^*^ is the ESS sex ratio. According to the theory of EES^3^,^22^, there are:


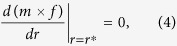


and


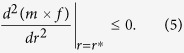


To avoid unnecessary technical details, in this following section, we focus only on the first condition—the equilibrium condition (Equation (4)).

Applying Equation (4) to Equation (3), we obtain the following ESR sex ratio as


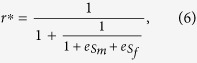


where 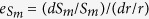 is the percentage change in male survival rate divided by the percentage change in sex ratio and 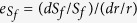 is the percentage change in female survival rate divided by the percentage change in sex ratio. They are similar to the elasticity concepts of economics, corresponding to the well-known *price elasticity of demand* and *price elasticity of supply*^20^. We therefore define 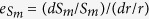 and 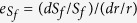 as the *sex ratio elasticity of male survival rate* (SRE-MSR) and the *sex ratio elasticity of female survival rate* (SRE-FSR), respectively.

From male’s perspective, the SRE-MSR is a measure of the responsiveness of male survival rate to a change in sex ratio. Similarly, from female’s perspective, the SRE-FSR measures the responsiveness of female survival rate to a change in sex ratio.

Obviously, from the definitions of SRE-MSR and SRE-FSR, the value of the SRE-MSR should be negative because male survival rate decreases with the increase of sex ratio, and the value of the SRE-FSR should be positive because female survival rate increases with the increase of sex ratio. The negative or positive sign only represents the direction of variation and the value represents the sensitive degree of survival rate to sex ratio^19^,^20^.

Furthermore, from the definition of sex ratio, the ESR must also satisfy *0* < r^*^ < *1*, (we only consider sexual organisms in this study). From Equation (6) and *0* < r^*^ < *1*, we have 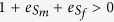; in the following, this constraint is maintained.

## Results

**Figure 1:**
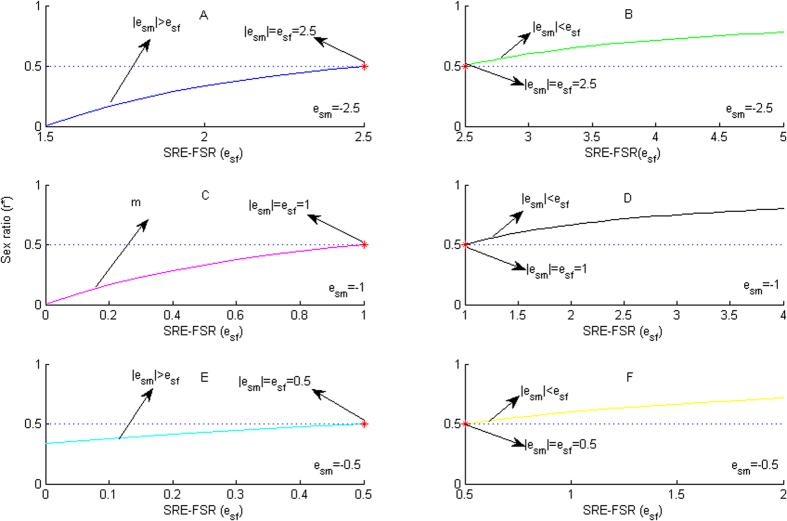
The relationship between the SRE-FSR and the ESR sex ratio.

From the above model constructions, we conclude the following results:

1. If we given 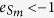, that is, the male survival rate is elastic, then
  1. When 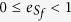 (the female survival rate is inelastic) and 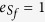 (the female survival rate is unitary elastic), the 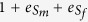 is less than 0, therefore, these cases is meaningless in our model.
  2. When 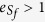 (the female survival rate also is elastic), (*i*) If 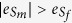, which means the sensitive degree of the male survival rate to sex ratio is greater than that of female, from Equation (6), we have r* < *1*/*2* as an ESR, i.e., the female-biased sex ratio is an ESR (Fig. 1A, blue line); (*ii*) If 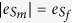, which means the sensitive degree of the male survival rate to sex ratio is equal to that of female, from Equation (6), we have r* = *1*/*2* as an ESR, *i.e.,* the unbiased sex ratio is an ESR (see the red star point of the Fig. 1A and B); (*iii*) If 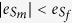, which means the sensitive degree of the male survival rate to sex ratio is less than the female, from Equation (6), we have r* > *1*/*2* as an ESR, *i.e.,* the male-biased sex ratio is an ESR (Fig. 1B, green line).
2. If we given 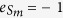, that is, the male survival rate is unitary elastic, then
  1. When 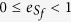 (the female survival rate is inelastic), i.e., 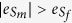, which means the sensitive degree of the male survival rate to sex ratio is greater than that of female, from Equation (6), we have r* < *1*/*2* as an ESR, i.e., the female-biased sex ratio is an ESR (Fig. 1C, magenta line);
  2. When 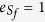 (the male survival rate is unitary elastic), i.e., 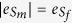 which means the sensitive degree of the male survival rate to sex ratio is equal to that of female, from Equation (6), we have r* = *1*/*2* as an ESR, *i.e.,* the unbiased sex ratio is an ESR (see the red star point of the Fig. 1C and Fig. 1D);
  3. When 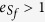 (the female survival rate is elastic), i.e., 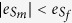, which means the sensitive degree of the male survival rate to sex ratio is less than the female, from Equation (6), we have r* > *1*/*2* as an ESR, *i.e.,* the male-biased sex ratio is an ESR (Fig. 1D, black line).
3. If we given 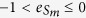, that is, the male survival rate is inelastic, then
  1. When 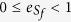 (the female survival rate is inelastic), (i) If 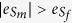, which means the sensitive degree of the male survival rate to sex ratio is greater than that of female, from Equation (6), we have r* < *1*/*2* as an ESR, i.e., the female-biased sex ratio is an ESR (Fig. 1E, cyan line); (ii) If 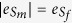, which means the sensitive degree of the male survival rate to sex ratio is equal to that of female, from Equation (6), we have r* = *1*/*2* as an ESR, *i.e.,* the unbiased sex ratio is an ESR (see the red star point of the Fig. 1E and F); (iii) If 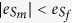, which means the sensitive degree of the male survival rate to sex ratio is less than the female, from Equation (6), we have r* > *1*/*2* as an ESR, *i.e.,* the male-biased sex ratio is an ESR (Fig. 1F, yellow line).
  2. When 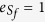 (the male survival rate is unitary elastic) and 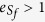 (the female survival rate is elastic), i.e., 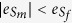, which means the sensitive degree of the male survival rate to sex ratio is less than the female, from Equation (6), we have r* > *1*/*2* as an ESR, *i.e.,* the male-biased sex ratio is an ESR (Fig. 1F, yellow line).

## Discussion

To study the evolution of sex ratio, previous models have separately dealt with how the mate competition among male offspring affects the ESR and how the resource competition among female offspring affects the ESR^2^,^3^,^8^,^9^. male offspring compete for one resources (such as space, mates) and female offspring compete for the other resources (such as nests) may occur simultaneous in a same patch, and these competitions could lead to the difference of the SRE-MSR and SRE-FSR^3^,^18^. However, as to our knowledge, how the SRE-MSR and SRE-FSR affect the ESR, which have never been addressed before^3^. The model described in this paper shows that if we assume that male offspring and female offspring compete for different resources, and male offspring compete for resources A (such as space, mates) and female offspring compete for resources B (such as nests), and there are two competitions occurring simultaneously the ESR depends on the SRE-MSR and SRE-FSR.

Our model firstly shows that if the intensity of the competition among male offspring for resources A equals to the intensity of the competition among female offspring for resources B, i.e. the sensitive degree of the male survival rate to sex ratio is equal to the female’s, the unbiased sex ratio is an ESR. In fact, when the mating is random in a large population and the resource competition among female is random (i.e. there are no competitive interactions between siblings), the intensity of the male competition is equal to the intensity of the female competition, in our model we predict that the ESR is ½. This conclusion is similar to Fisher’s equal investment theory, i.e., when mating is random, mothers favors equal investment into the two sexes, therefore, the ESR is ½^3^,^6^.

In addition, our model shows that if the competition among male offspring for resources A is more intense than the competition among female offspring for the resources B, i.e., the sensitive degree of the male survival rate to sex ratio is greater than the female’s, the ESR is the female-biased. This result is consistent with many empirical studies^3^,^16^,^23^. For example, for some *Arthropods* (such as, beetles, mites), in this species, a female and her brood occupy a gallery under bark, mating usually occurs before dispersal from the larval host. Obviously, *mates* can also be considered as a *resource* different from food and shelters. Therefore, the competition among male offspring for mates (resources A) is more intense than the competition among female offspring for nests (resources B), and strongly female biased sex ratio is observed^2^,^3^,^24^. To be noted that when 
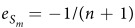
 and 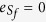, *i.e.*, 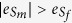, this result leads to the result of LMC^8^.

On the contrary, if the competition among male offspring for resources A is less intense than the competition among female offspring for resources B, we predict that the ESR is the male-biased. Moreover, the result of LRC is special case of our results, *i.e.*, if 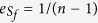 and 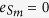 3,9. Moreover, this prediction is consistent with some empirical tests^3^. For example, in the African bush baby *Galago Crassicaudaus*, during the breeding season, female’s movement are restricted by the burden of raising offspring, consequently, the competition among female offspring for nests (as resources B) is more intense than the competition among male offspring for mates (as resources A), and so favors a male biased sex ratio reduce the competition among female offspring for resources B^9^.

Although using a simple sex ratio model and this study achieves several conclusions, there are still some limitations of the model used in this study. The model has disregarded a number of complicating factors, such as density dependence, disperse rate, the spatial structure. To some extent, adding these factors to the model may modify the conclusions reached in this study. We raise these issues to provoke further studies, not to mean that they are of secondary importance to a comprehensive theory of plant reproductive ecology.

## Additional Information

**How to cite this article**: Wang, Y. *et al*. Sex Ratio Elasticity Influences the Selection of Sex Ratio Strategy. *Sci. Rep.* **6**, 39807; doi: 10.1038/srep39807 (2016).

**Publisher's note:** Springer Nature remains neutral with regard to jurisdictional claims in published maps and institutional affiliations.
